# Outcomes and safety of concomitant topiramate or metformin for antipsychotics-induced obesity: a randomized-controlled trial

**DOI:** 10.1186/s12991-020-00319-x

**Published:** 2020-12-10

**Authors:** Congjie Wang, Wenjie Shi, Jianyang Xu, Chengbing Huang, Jiannan Zhu

**Affiliations:** 1 Department of Psychiatry, Huai’an No. 3 People’s Hospital, No.272 West Huaihai Rd., Huai’an, 223001 Jiangsu China; 2 Department of Neurology, Huai’an No. 3 People’s Hospital, No.272 West Huaihai Rd., Huai’an, 223001 Jiangsu China

**Keywords:** Topiramate, Metformin, Antipsychotics, Obesity

## Abstract

**Background:**

Although there are some existing data describing the usage of topiramate in patients with antipsychotic-induced obesity, study on its comparison with metformin is limited. This study aimed to explore the effectiveness and safety of concomitant topiramate on antipsychotic-induced obesity as well as its comparison with metformin.

**Methods:**

62 stabilized outpatients with antipsychotic-induced obesity were randomized into the topiramate group and the metformin group with 16-week treatment. The patients’ weight, body mass index (BMI), waist–hip ratio, and their side effects were assessed and compared. Intention-to-treat and completer analyses were performed. Meanwhile, covariance analysis was conducted to control the impact of the significant difference in BMI between the two groups.

**Results:**

The two groups had comparable characteristics, though their difference in baseline BMI was significant. (1) Intention-to-treat analyses: the random missing values were replaced using the last observation carried forward method when intention-to-treat analyses were conducted. Compared with the baseline, the weight, BMI, and waist–hip ratio in the topiramate group markedly decreased at each follow-up, whereas, in the metformin group, only waist–hip ratio significantly decreased at 4 weeks after treatment. Compared with the metformin, only weight and BMI in the topiramate group were significantly decreased at week 4 after treatment, and at week 8–16, weight, BMI and waist–hip ratio were remarkably declined. (2) Completer analyses: compared with the baseline, the weight, BMI, and waist–hip ratio in the topiramate group at week 4–16 were markedly decreased, whereas only waist–hip ratio with metformin was significantly decreased at week 4. Compared with the metformin, all BMI with topiramate were markedly decreased at week 4–16. Moreover, its weight and waist–hip ratio also were notably lowered at week 8. No significant differences in adverse events were found between the two groups.

**Conclusions:**

Topiramate, similar to metformin in reducing obesity as previously reported, also significantly reduced body weight, BMI, and waist–hip ratio in patients with antipsychotic-induced obesity and demonstrated well tolerance in psychiatric patients.

*Trial* *registration* The trial was registered at http://www.chictr.org.cn, and the number was ChiCTR-IPR-17013122.

## Introduction

Previous studies have linked some patient exposure to some second-generation antipsychotics (SGAs) to an increased risk for weight gain or obesity, hyperlipidemia, and impaired glucose metabolism, which properly was related to increased appetite/caloric intake and various receptors, hormones, and peptides have been implicated [1]. In particular, weight gain or obesity induced by SGAs may significantly increase both the risk of cardiovascular disease and mortality from cardiovascular disease [2, 3]. Moreover, the treatment of obesity caused by SGAs still remains a challenge. So far, there is no consensus on the exact effective and safe drug of weight loss, although many measures had been implemented to promote weight loss [4, 5].

Apart from behavioral interventions and switching to first generation antipsychotics or other SGAs, many medicines have been utilized to reduce weight gain or obesity induced by SGAs or non-pharmacological obesity in the past. For example, sibutramine, reboxetine, bupropion, orlistat, liraglutide, metformin, topiramate, and antagonism of the histamine 2 (H2) receptor, etc. were also reported to have significant effects on weight gain compared to placebo [6–9], whereas, some previous studies’ results were not consistent with each other, even there are some conflicting reports [10].

Metformin, a biguanide drug approved for treatment of type 2 diabetes, has been extensively studied for use of weight gain in the absence of diabetes in typically developing children [11, 12]. In adults or pediatric patients or adolescents, metformin may prevent or reverse or significantly attenuate weight gain associated with atypical antipsychotics [13–15], although some confound factors are difficult to be excluded, and its effects on body weight mostly were due to reduction in appetite rather than increases in calorie expenditure. Topiramate, a new antiepileptic drug, which is increasingly being used as a mood stabilizer in bipolar disorder, has also been used as adjuvant therapy for both the positive and negative symptoms of schizophrenia [16–18]. And it holds some promise as an adjunctive therapy for both overweight and treatment of psychotic symptoms [6]. Topiramate has the strongest mean weight loss compared with placebo, metformin, H2 receptor antagonists, and norepinephrine reuptake inhibitors in some reviews [6, 9, 19–21]. The effects of topiramate on patients with obesity caused by SGAs were clearly related to decreasing appetite and increasing satiety, but does not alter energy expenditure, perhaps through inhibition of carbonic anhydrase [6, 22], although the differences of topiramate on SGA-associated obesity varied in safety, efficacy, and response to therapy in the different subpopulations of patients with obesity [20–23].

In fact, the treatment of weight gain or obesity induced by antipsychotics still remains a significant challenge to psychiatrists, because there is relatively little evidence of specificity for pharmacological therapies to antipsychotic-induced obesity. Moreover, there is no consensus on the exact effective and safe drug of weight loss until now. Although some studies have evaluated the effects of topiramate or metformin on weight gain, most of them made the comparison with the placebo, and only one investigation with sibutramine [24].

Up to now, there is no research report on the direct comparison of topiramate with metformin on weight gain or obesity induced by SGAs in the real world. Therefore, we hypothesize that topiramate and metformin have the same effects on antipsychotic-related obesity, the aim of this study was to explore the effects of concomitant topiramate or metformin on obesity caused by some SGAs while keeping the patient’s illness stable and maintaining the original dosage of antipsychotic medications.

## Methods

### Study design and participants

This study was a 16-week, open design, randomized clinical trial, and all outpatients or inpatients with schizophrenia or affective disorder were Chinese Han population and from Huai’an No. 3 People’s Hospital in a naturalistic clinical setting from September, 2012 to December, 2016. The protocol for the study was granted approval from the scientific and ethics committee of Huai’an No. 3 People's Hospital (Ethical Review No. HASYkjk 2012-003). All patients continued to receive the original antipsychotic medications and community psychiatric care or did some light manual works after enrollment, and meanwhile, informed consent was obtained from patients or their legal guardians. Baseline information including vital signs, height, weight, waist and hip circumference, demographic characteristics, psychiatric history, medication history, and medication-related adverse effects was collected. All patients were called for follow-up every 4 weeks for at least 16 weeks after concomitant topiramate or metformin treatment. Patient’s weight, height, and waist and hip circumference (W–H ratio) were measured while dressed in light clothing without shoes on the same scale zeroed at each measurement and calculated by investigators at each follow-up visit. And blood routine, liver function, and electrocardiogram examination were also conducted as possible as we could at each follow-up visit.

All assessments were done by investigators, and the patients’ indicators must be accurately measured although investigators had known the patients’ therapy allocation at each follow-up visit. The measurement of height, weight, and waist and hip circumference was conducted by three psychiatric doctors trained with well consistency among them (correlation coefficient = 0.87–0.90).

Patients included in this study were diagnosed with schizophrenia or affective disorder based on Diagnostic and Statistical Manual of Mental Disorders: DSM-IV by reviewing clinic medical records. All outpatients or inpatients’ illness (*n* = 2) were in a stable condition, and the age varies from 15 to 55. Patients had been on antipsychotics for at least 6 months and gained more than 10% of their body weight on an antipsychotic before enrollment, and currently met the diagnostic criteria for obesity, which was specified as a BMI over 25 kg/m^2^ based on the Regional Office for the Western Pacific Region of WHO criteria [25].

Patients were excluded from the study if they had a history of intolerance or hypersensitivity to topiramate and metformin, or had a severe or unstable general medical illness, such as renal and liver function failure, or severe cardiovascular diseases. Patients who were pregnant and/or during lactation period were also not included.

**Fig. 1:**
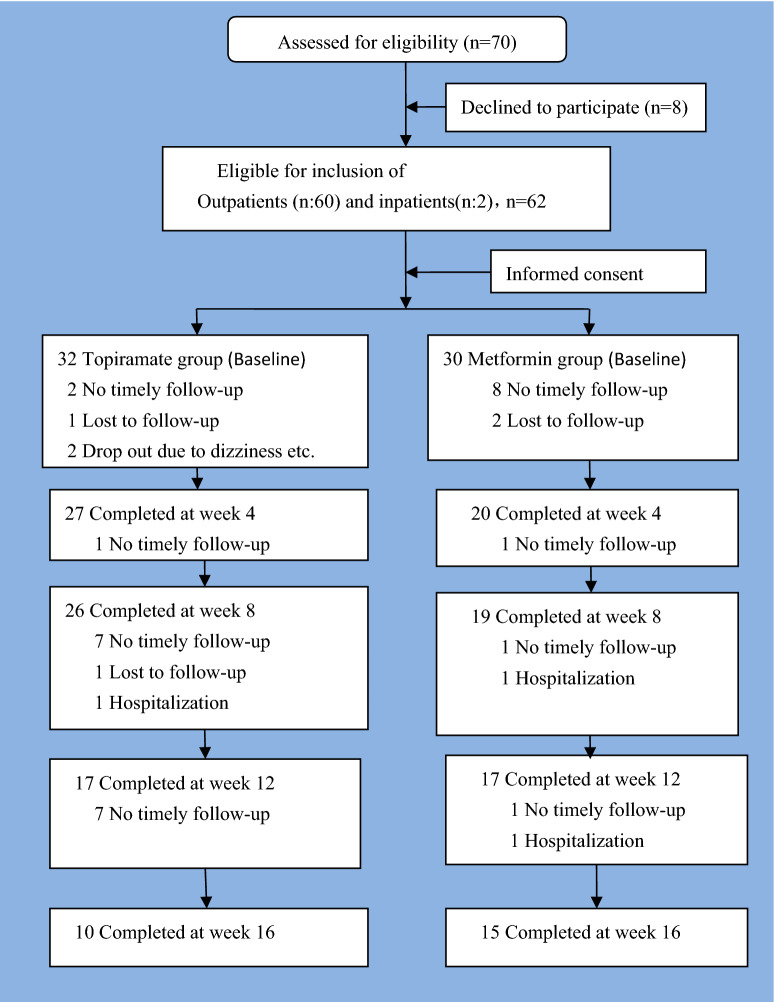
The diagram showing study design and flow of subjects in the study

70 cases of patients with schizophrenia or affective disorder with obesity induced by SGAs met with the inclusion criteria; 8 of them declined to participate the trial after the enrollment. Only 62 cases of obese outpatients (*n* = 60) and inpatients (*n* = 2) with schizophrenia or affective disorder in a stable condition of illness were included in this trail. Patients were randomly assigned to one of two groups using randomized table generated by online smart random A and B generator software (A represents the topiramate group; B represents the metformin group). Finally, 32 of 62 cases were assigned to the topiramate group, and 30 cases to the metformin group after enrollment. The outpatients in each group often were lost to follow-up or could not be followed up in time due to some reasons, such as the bad weather condition or personal reasons. The status of the enrollment, and the completed and withdrawn samples between the two groups are shown (see Fig. 1).

### Interventions

After patients were enrolled, all patients and caregivers only received brief counseling and information regarding the patients’ dietary management and physical exercise. The dosage of each antipsychotic the patients previously used remained unchanged as much as possible during the trial unless psychotic symptoms exacerbated or severe adverse events emerged. The treatment would be discontinued if investigators decided that a patient’s response was not adequate or the patient asked to be withdrawn from the trial due to severe adverse events or illness exacerbation.

It was assumed that the trial medication of topiramate and metformin could induce unpredictable side effects (e.g., augmented sedation, dizziness, or hypoglycemia), which might cause patients to drop‐out and make the results difficult/impossible to interpret. For that reason, a gradual increment in dosing of topiramate and metformin was chosen in this trial. Drug doses were chosen to minimize side effects, but retain clinically relevant therapeutic levels according to prior dose–response studies. Therefore, the initial dosage of topiramate was 50 mg twice daily, if tolerated, the maximum dose was 100–200 mg twice daily after 1 week, and the mean daily dose of topiramate was 190.63 ± 57.41 mg after 16-week treatment. The initial treatment dosage of metformin was 0.25 g twice daily, if tolerated, the maximum daily dose of metformin was 1.5 g after 1 week, and the mean daily dose of metformin was 0.67 ± 0.22 g after 16-week treatment. All patients received diet, drinking, or exercise counseling, and no other requirements were conducted.

### Outcome measurements

The primary outcome was measurement of weight, height, body mass index (BMI), and W–H ratio at each follow-up visit, which were measured every 4 weeks for 16 weeks. The BMI was calculated as weight in kilograms divided by height in meters squared, and W–H ratio was calculated as waist circumference divided by hip circumference.

The secondary outcomes were measurements of the patient’s liver function, blood routine, electrocardiogram at each follow-up visit, and adverse events were also monitored after concomitant topiramate or metformin every 4 weeks. Of course, the psychotic symptoms were also paid close attention to observe at each follow-up visit during the trial.

### Statistical analysis

The statistical analysis was carried out using SPSS 13.0 (SPSS Inc., Chicago, Illinois, USA). The categorical variables were tested using a Chi-square test, and the continuous variables were tested by means of a repeated measurement variance analysis before and after treatment in each group of concomitant topiramate or metformin, and independent samples *t* test between the two groups.

All missing data of two groups at each follow-up visit were random missing data by randomized analyses, and the missing data at week 4–16 of follow-up visit were replaced using simple imputation (last observation carried forward method, LOCF) when intention-to-treat (ITT) analyses was performed [26].

An intention-to-treat and completer analyses were performed between the two groups because of higher loss rate of follow-up. Covariance analysis was conducted to control the impact of the significant difference in baseline body mass index (BMI) when compared the patients’ weight, BMI, and waist–hip ratio at 4–16 weeks of follow-up visit between the two groups.

All statistical tests were two-tailed. The values represented as mean ± standard deviation (SD) at each follow-up visit. *P* value < 0.05 was considered statistically significant.

## Results

62 cases of patients with obesity entered two groups after 16 weeks of the trial. Of 62 cases of patients, there were 32 cases of outpatients or inpatients into the topiramate group and 30 cases into the metformin group respectively (see Fig. 1). It was listed in Table 1 related to patients’ demographic, clinical features at baseline, follow-up situation, and types of APS being used after the patients enrolled between the two groups.

**Table 1:** Comparison of patients’ demographic and clinical features at baseline and after the end of the trial between the two groups

Variables	Topiramate (*n*)	Metformin (*n*)	*χ*^2^/*t*	*P* value
*n*	32	30		
Gender (M_1_/F)	28/4	24/6	0.64	> 0.05
Age (years)	32.81 ± 9.11	31.87 ± 8.48	0.42	> 0.05
Marital status (M_2_/S_1_/D)	9 /22 /1	11 /16 /3	2.09	> 0.05
Occupation (F/J/E)	15 /13 /4	16 /8 /6	1.56	> 0.05
Level of education				
Primary education	15	13	0.44	> 0.05
Secondary education	15	16		
College and above	2	1		
Parental obesity				
No/yes	29/3	28/2	0.15	> 0.05
Diagnosis (S_2_/AD)	31/1	27/3	1.21	> 0.05
Inpatients/outpatients	2/30	0/30	1.94	> 0.05
The number of patients with/without loss of follow-up				
With/without loss of follow-up at week 4	5/27	10/20	2.65	> 0.05
With/without loss of follow-up at week 8	6/26	11/19	2.50	> 0.05
With/without loss of follow-up at week 12	15/17	13/17	0.08	> 0.05
With/without loss of follow-up at week 16	22/10	15/15	2.26	> 0.05
CPZ equivalent daily dose of all APS used during the trial	433.75 ± 215.57	370.17 ± 177.07	1.26	> 0.05
The main types of APS currently in use during the trial				
Clozapine	16	10	3.15	> 0.05
Olanzapine	6	6		
Quetiapine	2	5		
Risperidone	4	6		
Other APS, and etc.	4	3		
Daily dosage of clozapine during the trial (*n*: 16, 10)	229.69 ± 121.18	165 ± 114.99	1.35	> 0.05
Daily dosage of olanzapine during the trial (*n*: 6, 6)	10.0 ± 6.32	12.08 ± 5.10	0.63	> 0.05

*M*_*1*_ male, *M*_*2*_ married, *S*_*1*_ single, *D* divorced, *F* farmer, *J* jobless, *E* employee, *S*_*2*_ schizophrenia, *AD* affective disorder, *CPZ* chlorpromazine, *APS* antipsychotics

### Comparison of patients’ demographic and clinical features at baseline between the two groups

There were no statistically significant differences in gender, age, marital status, occupation, educational level, parents’ history of obesity, the diagnosis types of mental disorder, chlorpromazine (CPZ) equivalent daily dose of antipsychotics [27, 28], type of antipsychotics, and daily dosage of clozapine and olanzapine being used between the two groups. In addition, no remarkable differences were found in the type and the number of use of combined antipsychotics, affective stabilizers, antidepressants, chlorpromazine (CPZ) equivalent daily dosage of use of combined antipsychotics, and the number of outpatients and inpatients between the two groups. Therefore, the patients’ demographic, clinical characteristics, and the factors that continued to influence weight gain during the trial were comparable and matched between the two groups (see Tables 1, 2).

**Table 2:** Concomitant APS, affective stabilizers, and antidepressants between the two groups during the trial

Variables	Topiramate (*n*)	Metformin (*n*)	*χ*^2^/*t*	*P* value
The number of combined use of APS, affective stabilizers and antidepressants	18	27		
Types of combined use of APS, and etc.				
Clozapine	2	0	11.01	> 0.05
Olanzapine	0	3		
Quetiapine	2	1		
Risperidone	4	5		
Ziprasidone	2	1		
Aripiprazole	2	4		
Perphenazine	4	9		
Chlorpromazine	1	0		
Sulpiride	0	1		
Lithium carbonate	0	1		
Sodium valproate	0	1		
Antidepressants	1	1		
CPZ equivalent daily dose of combined use of APS	242.35 ± 198.26	342.50 ± 232.45	1.44	> 0.05

*APS* antipsychotics, *CPZ* chlorpromazine

### ITT analyses

The effects of concomitant topiramate or metformin on weight gain, BMI, and W–H ratio at each follow-up visit in each group or between groups before and after treatment.

All missing data at each follow-up visit were randomized missing data by randomized analysis, and the missing data at each follow-up visit were replaced using the last observation carried forward method (LOCF) when ITT analyses was performed.

**Fig. 2:**
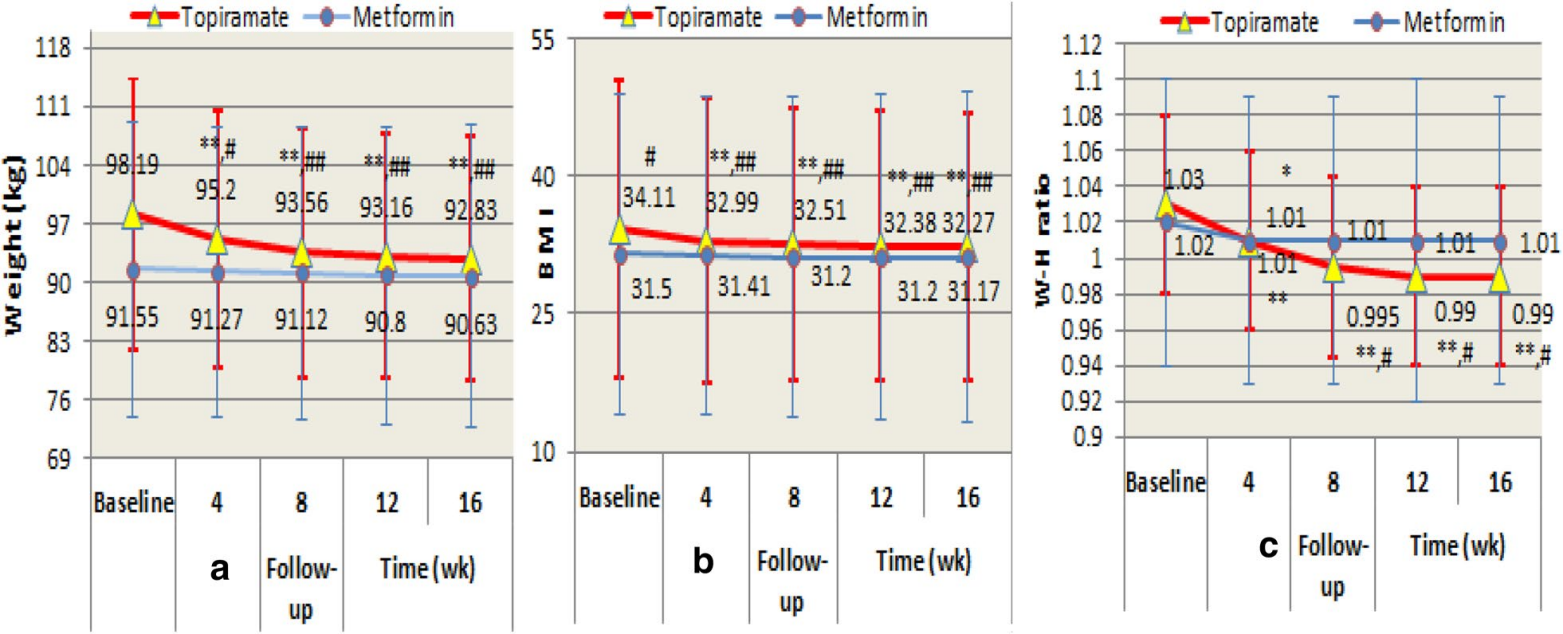
ITT analyses showed change in weight (**a**), BMI (**b**), and W–H ratio (**c**) during treatment: compared with baseline: *P < 0.05, **P < 0.001; with metformin: ^#^*P* < 0.05, ^##^*P* < 0.01

Compared with the baseline in each group, the body weight, BMI and most waist–hip ratio at each follow-up visit markedly decreased (all *P* value < 0.001), only waist–hip ratio at week 4 of follow-up visit significantly decreased in the topiramate group (*t* = 2.63, *P* < 0.05), while no differences of weight loss, BMI, and waist–hip reduction ratio were found at each follow-up visit compared with baseline in the metformin group (see Fig. 2).

Compared with the metformin group, only weight and BMI at week 4 of follow-up visit in the topiramate group significantly declined (*t* = 4.38, *P* < 0.05; *t* = 9.28, *P* < 0.01), but there was no difference in waist–hip ratio at 4 weeks of follow-up visit between the two groups. The reduction of weight, BMI, and waist–hip ratio at 8, 12, and 16 weeks of follow-up visit in the topiramate group were all remarkably more than that in the metformin group (*P* < 0.05, or *P* < 0.001) (see Fig. 2).

### Completer analyses

Effects of combined treatment with topiramate or metformin on weight, BMI and WHR of patients who completed the entire trial at each follow-up in each group or between the two groups before and after concomitant treatment.

Figure 1 shows the status of completed this trail at each follow-up visit. Only 10 cases of patients in the topiramate group and 15 cases of patients in the metformin group at week 16 of follow-up visit were included in the completer analyses. The loss rate of follow-up was similar at weeks 4–16 of follow-up visit between the two groups (all *P* > 0.05) (see Table 1).

**Fig. 3:**
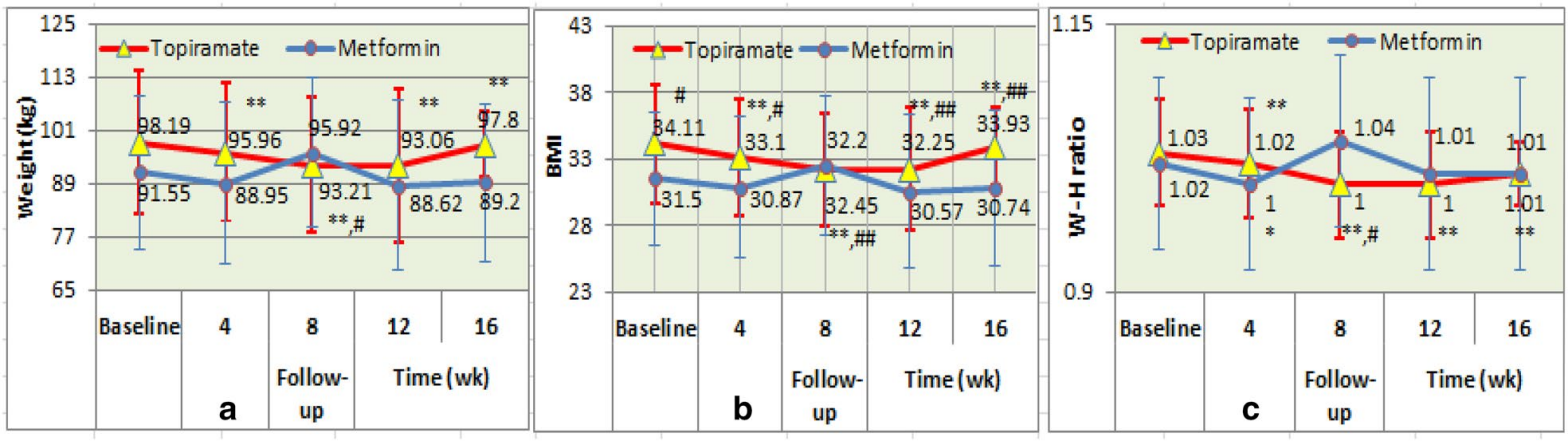
Completer analyses showed change in weight (**a**), BMI (**b**), and W–H ratio (**c**) during treatment: compared with baseline: *P < 0.05, **P < 0.001; with metformin: ^#^*P* < 0.05

*Compared with the baseline in each group*, statistically significant reductions of weight, BMI, and W–H ratio from weeks 4 to 16 of follow-up were found in the topiramate group (all *P* value < 0.001). However, only statistically remarkable reduction of W–H ratio at week 4 of follow-up visit was found in the metformin group (*P* < 0.05), there were no differences in weight loss and BMI reduction at week 4 of follow-up visit, and the differences in three main measurements at other follow-up visit were also not found in the metformin group (see Fig. 3a–c).

*Compared with the metformin group*, there was significant difference in reduction of BMI from weeks 4 to 16 of follow-up visit in the topiramate group (*P* < 0.05–0.01), and there were also marked weight loss and W–H ratio reduction at week 8 of follow-up visit in the topiramate group, but the differences in above three measurements at other follow-up visit were not found between the two groups (see Fig. 3a–c).

### Adverse events

There were 2 cases of outpatients with both dizziness and poor appetite, one case of patient with diarrhea, abnormal liver function and exacerbation of illness in the topiramate group during the trial. Only 2 cases of outpatients with abnormal liver function or 2 cases with exacerbation of illness were reported in the metformin group after 16-week clinical trial, respectively. There were no significant differences in above adverse events between the two groups, no other intolerable adverse events, such as severe organic impairment, were found between the two groups during the trial (*P* > 0.05). One patient presented with worsening psychiatric symptoms in the topiramate group and two patients in the metformin group were found after the end of this study, no significant difference in worsening psychiatric symptoms was found between the two groups.

## Discussion

Although some previous research suggested that metformin may have robust role of weight loss, this study further indicated that topiramate was significantly superior, at least similar, to metformin in managing established weight gain or obesity caused by some second-generation antipsychotics by ITT and completer analyses in the case of keeping the patient’s illness stable and maintaining the original antipsychotic treatment. The benefits from weeks 4 to 16 of follow-up visit after treatment of concomitant topiramate were emerged whether in terms of weight loss or BMI and waist–hip ratio reduction both by ITT analysis and completion analysis. It also indicated that topiramate has a faster and better therapeutic effect on obesity caused by SGAs. Whereas, the effects of concomitant metformin on obesity associated with atypical antipsychotics were not significant, and only waist–hip ratio reduction at week 4 of follow-up visit remarkably decreased compared with the baseline by ITT analyses and completer analyses. The results associated with topiramate were consistent with most studies’ reports and meta-analyses reviews [6, 19, 29–31]. Whereas, the results related to metformin in this study were not completely consistent with some previous studies’ reports, especially in terms of weight loss [13, 14, 32]. This may be associated with lower dosage of metformin being used in this study. Topiramate has been approved as a weight loss drug with concomitant use of phentermine by the US Drug Administration (FDA) [33], and most previous studies’ results also revealed that topiramate has a significant antipsychotic effect and weight loss, although not all studies’ results related to the effects of topiramate on psychosis are consistent [34, 35].

The comparison between concomitant use of topiramate and metformin revealed that topiramate has a robust therapeutic effect on obesity induced by some SGAs during the vast majority of follow-up visit in this trial. This was consistent with most previous meta-analyses results [6, 19]. Nevertheless, it was not consistent with Ellinger’s review [10], which indicated the use of metformin resulted in greater weight loss than topiramate. This perhaps was associated with Ellinger’s review without a meta-analyses and statistical test.

It should be noted that, during the trial, the weight at week 8 elevated transitorily in the metformin group, and it did so at week 16 in the topiramate group. All this suggested that outpatients in the real world often cannot follow the experimental requirements, especially in outpatients with mental disorders. They even frantically failed to control their diet and refused to take part in all activities, particularly during the onset of certain mental illnesses.

The mechanism of weight loss for topiramate was mainly associated with its decreasing appetite and increasing satiety, but did not change energy expenditure, probably by means of inhibition of carbonic anhydrase [6]. In addition, topiramate itself also played a role in schizophrenia, which was considered to be mediated by antagonism of glutamate-caused excitotoxicity at the kainic acid (KA)/alpha-amino-3-hydroxy-5-Methylisox-azole-4-propionic acid (AMPA) glutamate receptors [36]. Altered levels of free glutamate, in which topiramate serves as an antagonist, have been found in schizophrenic patients compared with healthy controls [22].

There were no significant differences regarding the loss rate of follow-up at each follow-up visit, the incidence of adverse events, and the number of recurrence of the original mental disorders between the two groups during the trial, which suggested that the tolerance and safety of topiramate and metformin were similar when they were utilized to treat weight gain or obesity induced by SGAs. However, the results of this study were not exactly consistent with some previous reviews or study results [6, 10]. It was perhaps related to the absence of statistical tests about adverse events in these reports.

The use of topiramate, of course, also has some rare adverse events, such as dizziness, diarrhea, and inappetence, which should not be ignored, although there were no difference between the two groups. However, the diarrhea and inappetence would be significantly alleviated after reducing the dose of topiramate. To be sure, the recurrence or worsening of illness in some outpatients was mostly due to non-compliance for the antipsychotic treatment during the trial, instead of associating with the concomitant use of topiramate or metformin, although it was not consistent with Choi et al.’s report [37].

As to the effects of topiramate on maintaining stability in schizophrenia or affective disorders, in this study, only three cases of psychiatric symptoms worsen were found in the topiramate and metformin group at the end of this study, and no significant difference was found between the two groups. Therefore, there were no enough evidence to prove that topiramate might stabilize the illness of schizophrenia and affective disorders according to this results.

There are some limitations to this study. First, the dosage of metformin in this study was relatively lower than that in other previous studies. This may be one of the reasons why metformin reversed antipsychotics-induced obesity was inferior to topiramate. Secondly, the number of cases in the study was relatively small. And the number of completer was also gradually decreased with the increase of follow-up time, which needs more concerns in future research, although there was no remarkable difference in the loss rate of follow-up between the two groups.

In summary, in the treatment of weight gain or obesity induced by some SGAs, topiramate holds great promise for reducing weight gain, BMI, and waist–hip ratio compared to metformin, although metformin may be a more appropriate agent in people with obesity or diabetes. Of course, the ideal approach to weight loss should be also highly individualized, identifying appropriate medications, and behavioral intervention.

## Conclusion

The topiramate, as an adjunctive therapy, is similar to metformin in reducing obesity as previously reported. It also significantly decreases antipsychotic-induced obesity and waist–hip ratio in patients with schizophrenia, affective disorder, etc., and has a better acceptability and fewer adverse effects.

## Abbreviations

BMI: Body mass index
W–H ratio: Waist–hip ratio
LOCF: Last observation carried forward method
SGAs: Second-generation antipsychotics
H2: Histamine 2
ITT: Intention-to-treat
